# Embolization of multiple splenic artery aneurysms in a patient with hypersplenism due to portal hypertension: a case report

**DOI:** 10.1590/1677-5449.202301392

**Published:** 2024-08-09

**Authors:** Lucas Victoy Guimarães Zengo, Maria Vitoria Bandeira Liebich, Larissa Rossi, Giuliana Rossato Biezus, Jeferson Freitas Toregeani, Jong Hun Park

**Affiliations:** 1 Centro Universitário Fundação Assis Gurgacz – FAG, Cascavel, PR, Brasil.; 2 Faculdade de Ciências Médicas da Santa Casa de São Paulo, São Paulo, SP, Brasil.

**Keywords:** aneurysms, splenic artery, cirrhosis, hypersplenism, portal hypertension, embolization

## Abstract

Aneurysms of the splenic artery are the third most common type of intra-abdominal aneurysms and the most common type of visceral aneurysms. Portal hypertension is a significant risk factor for development of these aneurysms. We report the case of a white, female, 52-year-old patient with multiple splenic artery aneurysms and hypersplenism secondary to portal hypertension and cirrhosis. Abdominal angiotomography identified six splenic aneurysms. In this scenario, an endovascular intervention was scheduled to conduct embolization using controlled release coils and Onyx™ embolization agent. The three largest aneurysms were treated. Control angiographs showed good exclusion of the aneurysms. The endovascular technique therefore proved to be a good choice considering the patient’s comorbidities and blood disorders. In this case, the procedure was successful. There were no immediate or long-term complications. The patient recovered well and is in clinical follow-up.

## INTRODUCTION

Splenic artery aneurysms are considered the third most common type of intra-abdominal aneurysm, after aortic and iliac aneurysms,^1^ and are considered the most common type of visceral aneurysms, with a prevalence of 60% of visceral aneurysms^2^ and an incidence of 0.1% in the general population.^3^ There is a predilection for females, at a proportion of 4:1.^4^ They are generally asymptomatic, small (with diameters from 2 to 4 cm), saccular, and located in the distal mid third of the splenic artery.^5^ Rupture is a catastrophic event and may manifest with hypotension and pain in the upper left quadrant, radiating to the subscapular area.^6^

Intervention options include traditional open surgery, laparoscopic surgery, and endovascular surgery. Intervention is recommended for patients with symptomatic aneurysms, women of reproductive age with all sizes of aneurysms, and patients with asymptomatic aneurysms of 3 cm or larger.^7^ W report the case of a female patient with multiple splenic artery aneurysms and hypersplenism secondary to portal hypertension who was treated using endovascular techniques.

The research protocol was approved by the Research Ethics Committee under Ethics Appraisal Submission Certificate number 70087323.3.0000.5219 (approval decision number 6.156.226). The recommendations set out in the CARE (CAse REport) guidelines were followed in writing this report.

## PRESENTATION OF THE CASE

The patient was a 52year-old, white skinned female with a long-term history of abdominal pains and two recent admissions for hepatic encephalopathy. Physical examination found hepatosplenomegaly. Comorbidities included cirrhosis, portal hypertension, hypersplenism, and blood disorders (thrombocytopenia, elevated international normalised ratio, and prolonged activated partial thromboplastin time). Prior history included acute myocardial infarction 12 years previously, heart failure, and hypothyroidism.

**Figure 1:**
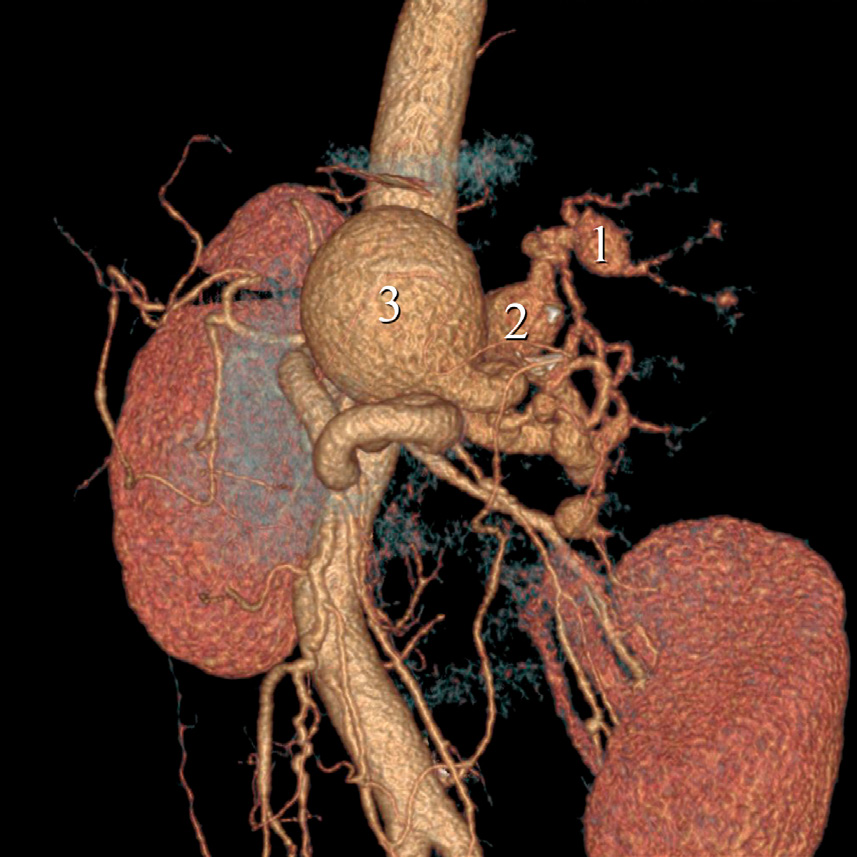
Angiotomography with three-dimensional reconstruction, showing the larger aneurysms.

The patient was referred to the vascular surgeon with abdominal ultrasonography findings including a large aneurysm of the splenic artery. The investigation was continued with abdominal angiotomography, which revealed multiple splenic artery aneurysms. Three of these aneurysms were small, distal to the splenic artery, located at the inferior pole of the spleen. One of the larger ones, with a diameter of about 2 cm (aneurysm 1), was distal and superior. The second largest had diameters of 3.4 x 2.4 cm (aneurysm 2) and was located medial-distally. The largest aneurysm had diameters of 5.1 x 4.7 cm (aneurysm 3) and was located at the mid third of the splenic artery. Figure 1 shows the three aneurysms (1, 2, and 3) as seen in the abdominal angiotomography three-dimensional reconstruction image.

The patient underwent a series of assessments with a cardiologist, a hematologist, and a digestive apparatus surgeon. The possibility of splenectomy was discussed, but was contraindicated by the surgeon because of the patient’s multiple comorbidities. However, all her vaccinations were fully up to date, in case elective splenectomy were to become necessary. The team therefore decided on endovascular management with embolization of the splenic aneurysms. The day before the procedure, the patient was given vitamin K and a platelet transfusion.

At the start of the procedure, a hydrophilic guidewire and a 6F long introducer were used to puncture the right brachial artery and a vertebral catheter and stiff 0.035" guidewire were advanced via the axillary artery, subclavian artery, aortic arch, and descending aorta to the celiac trunk, under angiographic control.

**Figure 2:**
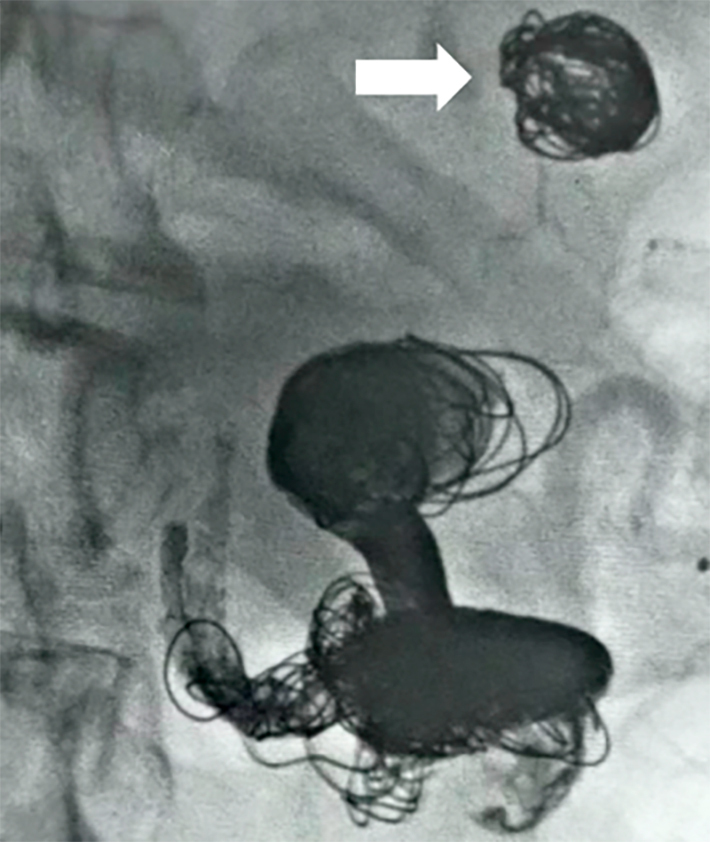
Embolization of the first aneurysm (2 x 2 cm).

Selective angiography of the splenic artery revealed multiple aneurysms. Next, a Traxcess 0.014" microguide and a Rebar 18 microcatheter were advanced across the largest aneurysm (5.1 x 4.7 cm) to locate the distal superior aneurysm, with a diameter of around 2 cm (aneurysm 1). The first step of the procedure therefore consisted of embolization of aneurysm 1. This was achieved by controlled detachment of Axium Frame 18 x 50, 12 x 40, and 14 x 50 microcoils using a mechanical detachment device. Control angiography then showed good exclusion of aneurysm 1 (Figure 2).

**Figure 3:**
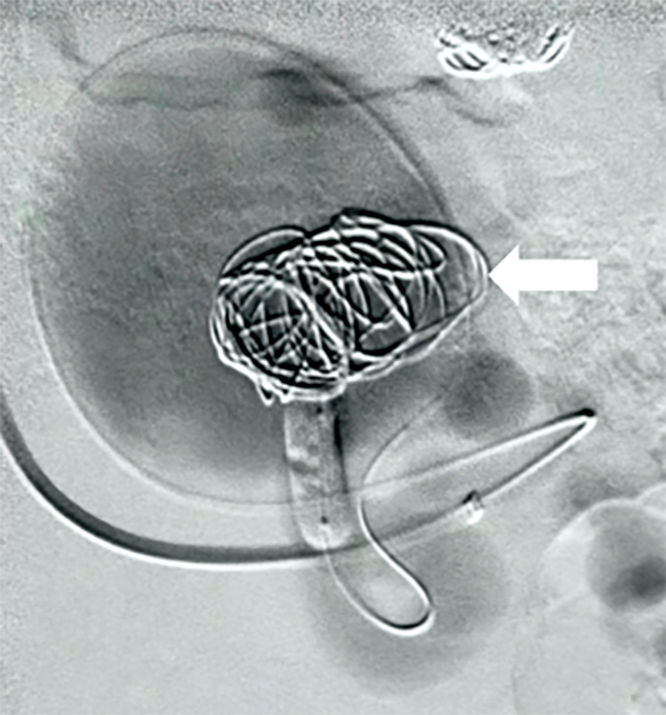
Embolization of the second aneurysm (3.4 x 2.4 cm).

Next, an Xpedion 0.010" guide, an Echelon 10 microcatheter, and a Hyper Glide 5 x 20 balloon catheter were positioned for blood flow control. The next step was to embolize aneurysm 2 (3.4 x 2.4 cm) with Axium Frame 20 x 50, 18 x 50, and 16 x 40 microcoils. Additionally, Onyx™ 18 embolization agent was also used (3 mL, two vials), since this aneurysm was larger than the first. Once more, control angiography demonstrated good exclusion of the aneurysm (Figure 3).

After angiography of the splenic artery, three small distal aneurysms were found, located at the inferior pole of the spleen. Multiple attempts were made to advance microcatheters and microguides, but it was not technically possible to embolize these aneurysms because of tortuosity of the splenic artery and successive failures to advance the catheter across the aneurysms. The decision was therefore taken to leave these aneurysms untreated.

**Figure 4:**
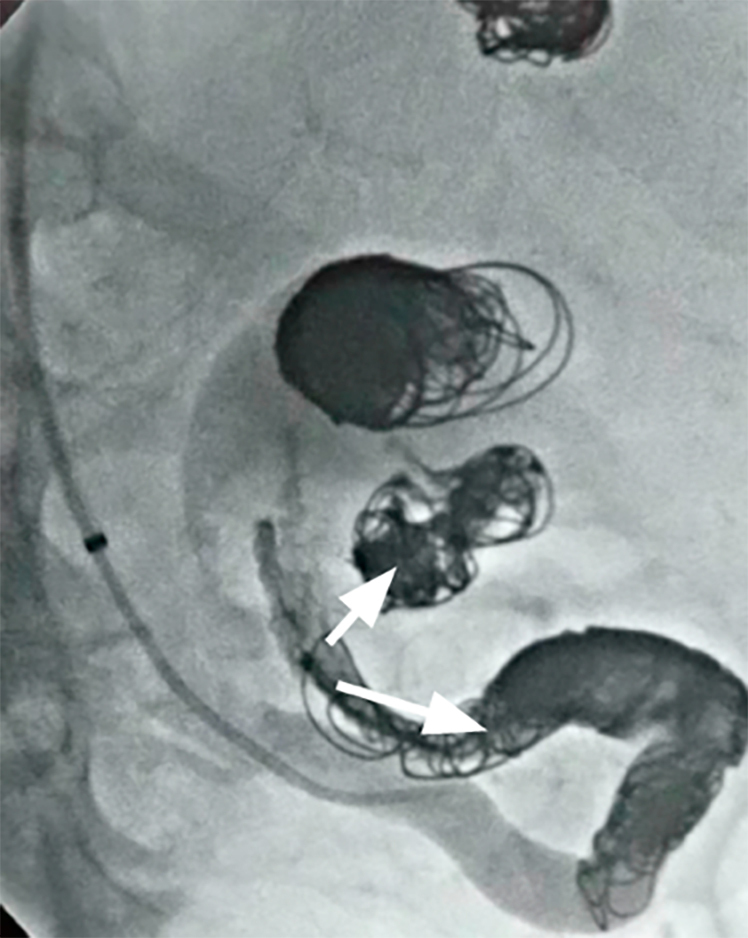
Embolization of the largest aneurysm (5.1 x 4.7 cm) using the trapping technique.

The last important step in the procedure was embolization of aneurysm 3 (5.1 x 4.7 cm). The endovascular technique of *“*trapping” was employed because the aneurysm was too large for embolization. A 5 Fr Simmons 2 curved catheter was introduced via the right common femoral artery, advancing catheter and guidewire up to the abdominal aorta. Next, the Rebar 18 microcatheter was advanced. In the artery distal of the aneurysm, Axium Frame 10 x 40, 14 x 40, and 5 x 20 microcoils were detached and a Hyper Glide 5 x 20 balloon catheter was advanced for blood flow control with Onyx™ 18 embolization agent (3 mL, two vials). Control angiography showed good sealing in the distal artery. Next, a 12 x 60 balloon was advanced past the lesion to temporarily occlude the splenic artery proximal of the aneurysm to facilitate release of the coils and the embolization agent and prevent migration into the aneurysm sac. Finally, Axium Frame 12 x 50, 6 x 20, and 5 x 20 microcoils and Ruby Coil 16 x 60, 8 x 60, and 4 x 35 microcoils were detached into the artery proximal of the aneurysm followed by embolization with Onyx (Figure 4).

Control angiography showed absence of blood flow into aneurysms 1 and 2 and very discrete blood flow through the afferent artery of aneurysm 3. After several attempts with coils and embolization agent, it was decided to wait for the effect of the heparin to recede and monitor for possible thrombosis. Perfusion to the pancreas was preserved, since the dorsal and greater pancreatic arteries were patent. There was no splenic infarction.

The patient was kept in the intensive care unit for clinical management. She suffered hematoma at the brachial access, which was corrected with simple arteriorrhaphy, and did not suffer any complications related to the femoral access. She recovered well during the postoperative period, requiring constant adjustment of coagulation factors and platelets. She was discharged on the sixth postoperative day with no complaints of abdominal pains. After the procedure, the patient is clinically stable and her platelet series has stabilized. The patient have been followed up for 2 years. Duplex Scan have shown exclusion of aneurysms 1 and 2, with 90% of exclusion of aneurysm number 3, with little reperfusion at the neck, near splenic artery. Last CT Scan showed no signs of expansion.

## DISCUSSION

The literature shows that the risk factors for development of aneurysms of the splenic artery include arterial hypertension, atherosclerotic disease, cirrhosis, portal hypertension, liver transplantation, female sex, pregnancy, and multiparity.^8^ Among these factors, portal hypertension is a significant risk factor for development of splenic artery aneurysms^9^ and the risk of aneurysm rupture is higher among patients with a history of portal hypertension than in those without this clinical history (56% vs. 17%, respectively).^3^ The patient in our case had almost all of the risk factors listed above. For this reason, there was a large risk of aneurysm rupture and splenic infarction.

Although the exact mechanism of aneurysmal dilatation is unknown, some authors have identified contributory factors, such as a preexisting weakening of the artery wall, rupture of the internal elastic lamina, reduced elastic fibers among multiparous women, and hormonal changes during pregnancy. These conditions may be present in patients with liver disease and portal hypertension with splenomegaly, among whom the incidence of splenic artery aneurysms is close to 20%.^10^

It is very important to emphasize the possibility of complications over the long term. Of these, splenic infarction can occur in the short term, but did not in this case because the patient had good collateral circulation via the short gastric arteries. According to the literature, splenic infarction and reperfusion of the aneurysm occur in 5 to 20% of patients.^11,12^ Over the medium term, splenic abscess and another complication, pancreatic necrosis, are both possible. In our case, control arteriography at the end of the procedure showed the dorsal and greater pancreatic arteries were patent, with good perfusion to the pancreas. In order to monitor for reperfusion of the aneurysm, annual follow-up is necessary with abdominal computed tomography or magnetic resonance.^11^

Possible interventions include traditional open surgery, laparoscopic surgery, and endovascular techniques. Intervention is recommended for patients with symptomatic aneurysms, women of reproductive age with aneurysms, and patients with asymptomatic aneurysms of 3 cm or larger.^7^ Surgical procedures include ligature or resection of proximal lesions or those at the mid portion of the splenic artery. Bypass surgery is not necessary since the short gastric arteries provide collateral blood flow to supply distal perfusion of the spleen. However, the “trapping” technique offers a minimally invasive alternative to surgical ligature. In this procedure, the aneurysm is trapped and excluded from the circulation by placing coils in the splenic artery both distal and proximal to the aneurysm and verifying that no collateral blood flow to the aneurysm remains.^6^

Splenic artery aneurysms tend to be ideal candidates for embolization because of the collateral supply from the short gastric arteries. Reported success rates vary from 90 to 100%.^12-14^ For aneurysms of the proximal splenic artery and medial vessels, stenting can be used to maintain patency of the main artery, but tortuosity of the splenic artery can make placement and deployment of stents difficult.^12,15^

## CONCLUSIONS

We described the case of a 52-year-old patient with cirrhosis, portal hypertension, and hypersplenism who presented with multiple splenic artery aneurysms. The endovascular technique was a good option in view of the patient’s comorbidities and blood disorders. In this case, the procedure was successful. Despite bleeding on the right arm access, after all, patient recovered well and have been followed up for 2 years. Embolization was successfull in aneurysms 1 and 2 ant trapping technique had satisfactory result on number 3 aneurysm.
