# Supplementary material for: Embolization of multiple splenic artery aneurysms in a patient with hypersplenism due to portal hypertension: a case report
Source: J Vasc Bras. 2024 Aug 9;23:e20230139. doi: 10.1590/1677-5449.202301392 (PMC11404771; doi:10.1590/1677-5449.202301392)
Supplement: Translation

# Translation: Embolização de múltiplos aneurismas de artéria esplênica em paciente com hiperesplenismo por hipertensão portal: relato de caso

**DOI:** 10.1590/1677-5449.202301391

**Authors:** Lucas Victoy Guimarães Zengo, Maria Vitoria Bandeira Liebich, Larissa Rossi, Giuliana Rossato Biezus, Jeferson Freitas Toregeani, Jong Hun Park

**Affiliations:** 1 Centro Universitário Fundação Assis Gurgacz – FAG, Cascavel, PR, Brasil.; 2 Faculdade de Ciências Médicas da Santa Casa de São Paulo, São Paulo, SP, Brasil.

**Keywords:** aneurismas, artéria esplênica, cirrose, hiperesplenismo, hipertensão portal, embolização

## Abstract

Os aneurismas de artéria esplênica são o terceiro tipo mais comum de aneurismas intra-abdominais, sendo considerados o tipo mais comum de aneurismas viscerais. A hipertensão portal é um fator de risco significativo para o seu desenvolvimento. Relatamos o caso de uma paciente do sexo feminino, branca, de 52 anos, com múltiplos aneurismas de artéria esplênica com hiperesplenismo por hipertensão portal e cirrose. Por meio da angiotomografia abdominal, foram identificados seis aneurismas esplênicos. Nesse contexto, optou-se por intervenção endovascular por meio da embolização com molas de destaque controlado e material embolizante Onyx™. Os três aneurismas maiores foram tratados. As angiografias de controle mostraram boa exclusão dos aneurismas. Portanto, a técnica endovascular foi uma boa opção devido às comorbidades e às discrasias sanguíneas apresentadas. Neste caso, o procedimento foi bem-sucedido. Não houve intercorrências imediatas ou complicações a longo prazo. A paciente evoluiu bem, seguindo em acompanhamento clínico.

## INTRODUÇÃO

Os aneurismas de artéria esplênica são considerados o terceiro tipo mais comum de aneurismas intra-abdominais, após os aneurismas aórticos e ilíacos^1^ , sendo considerados o tipo mais comum de aneurismas viscerais, com prevalência de 60% entre os aneurismas viscerais^2^ e incidência de 0,1% na população geral^3^ . Há predomínio do sexo feminino na proporção de 4:1^4^ . Geralmente são assintomáticos, pequenos (diâmetro de 2 a 4 cm), saculares e localizados entre os terços médio e distal da artéria esplênica^5^ . A ruptura é um evento catastrófico e pode se manifestar com hipotensão e dor no quadrante superior esquerdo com irradiação para a região subescapular^6^ .

As opções para intervenção incluem cirurgia aberta tradicional, cirurgia laparoscópica e cirurgia endovascular. Os aneurismas sintomáticos, aneurismas em mulheres com idade fértil e aneurismas maiores que 3cm devem ser tratados cirurgicamente^7^ . Nesse contexto, relatamos o caso de uma paciente com múltiplos aneurismas de artéria esplênica, apresentando hiperesplenismo por hipertensão e submetida ao tratamento pela técnica endovascular.

O projeto de pesquisa foi aprovado pelo Comitê de Ética em Pesquisa com Certificado de Apresentação de Apreciação Ética número 70087323.3.0000.5219 (número do parecer aprovado: 6.156.226). Foram seguidas as recomendações das diretrizes CARE (CAse REport guidelines) para elaboração do relato de caso.

## APRESENTAÇÃO DO CASO

Tratou-se de uma paciente do sexo feminino, de 52 anos, branca e com história de dor abdominal de longa data e dois internamentos recentes devido à encefalopatia hepática. Ao exame físico, apresentou hepatoesplenomegalia. Entre as comorbidades, apresentou cirrose, hipertensão portal, hiperesplenismo e discrasias sanguíneas (plaquetopenia e relação normatizada internacional e tempo de tromboplastina parcial ativada alargados). A história pregressa incluía infarto agudo do miocárdio há 12 anos, insuficiência cardíaca e hipotireoidismo.

A paciente foi encaminhada ao cirurgião vascular com achado de ultrassonografia abdominal, a qual evidenciou um grande aneurisma de artéria esplênica. Para melhor investigação, foi solicitada uma angiotomografia de abdome, sendo observados múltiplos aneurismas de artéria esplênica. Destes, três aneurismas eram pequenos, situados distalmente à artéria esplênica, no pólo inferior do baço. Outro, com cerca de 2 cm de diâmetro (aneurisma 1), situava-se distal e superiormente. O segundo maior apresentava diâmetro de 3,4 x 2,4 cm (aneurisma 2), com localização médio-distal. O maior aneurisma apresentava diâmetro de 5,1 x 4,7 cm (aneurisma 3), situado no terço médio da artéria esplênica. A Figura 1 mostra os três aneurismas (1, 2 e 3), visualizados na reconstrução tridimensional da angiotomografia de abdome.

**Figura 1:** Angiotomografia com reconstrução tridimensional evidenciando aneurisma maior.

A paciente foi submetida a uma série de avaliações com cardiologista, hematologista e cirurgião do aparelho digestivo. Cogitou-se a hipótese de esplenectomia, fato contraindicado pelo cirurgião devido às múltiplas comorbidades da paciente. Não obstante, a paciente apresentava o esquema vacinal completo, caso fosse necessária a esplenectomia eletiva. Dessa forma, optou-se pelo manejo endovascular por meio da embolização dos aneurismas esplênicos. Na véspera do procedimento, a paciente recebeu vitamina K e transfusão plaquetária.

No início do procedimento, por meio de fio guia hidrofílico e introdutor 6 F longo, puncionou-se a artéria braquial direita, passando o cateter vertebral e guia *stiff* 0,035" pela artéria axilar, artéria subclávia, arco aórtico e aorta descendente até o tronco celíaco, realizando o controle angiográfico.

A angiografia seletiva da artéria esplênica possibilitou a visualização de múltiplos aneurismas. Em seguida, fez-se a passagem do microguia Traxcess 0,014" e microcateter Rebar 18 através do aneurisma maior (5,1 x 4,7 cm), localizando o aneurisma distal superior, a cerca de 2 cm de diâmetro (aneurisma 1). Por conseguinte, a primeira etapa do procedimento consistiu na embolização do aneurisma 1. Para isso, fez-se a liberação controlada de micromolas Axium Frame de 18 x 50, 12 x 40 e 14 x 50, com destacador mecânico. A seguir, a angiografia de controle evidenciou boa exclusão do aneurisma (Figura 2).

**Figura 2:** Embolização do primeiro aneurisma (2 x 2 cm).

Depois, fez-se a passagem do guia Xpedion 0,010", microcateter Echelon 10 e cateter balão Hyper Glide 5 x 20 para controle do fluxo sanguíneo. Então, a etapa seguinte foi a embolização do aneurisma 2 (3,4 x 2,4 cm) com micromolas Axium Frame de 20 x 50, 18 x 50 e 16 x 40. Ademais, pelo fato de ser um aneurisma maior que o primeiro, optou-se por acrescentar o agente embolizante Onyx™ 18 (3 mL, dois frascos). Igualmente, a angiografia de controle demonstrou boa exclusão do aneurisma (Figura 3).

**Figura 3:** Embolização do segundo aneurisma (3,4 x 2,4 cm).

Após uma angiografia de artéria esplênica, foram identificados três pequenos aneurismas distais, localizados no pólo inferior do baço. Houve múltiplas tentativas de passagem de microcateteres e microguias, porém, tecnicamente, não foi possível realizar a embolização devido à tortuosidade da artéria esplênica e à evasão repetida do cateter à passagem pelos aneurismas. Portanto, optou-se pelo não tratamento dessas lesões.

A última etapa importante do procedimento foi a embolização do aneurisma 3 (5,1 x 4,7 cm). Para tal passo, optou-se pela técnica de *trapping* endovascular, visto que o aneurisma era demasiadamente grande para embolização. Foram introduzidos cateteres de Simmons curva 2 de 5 Fr pela artéria femoral comum direita, navegando o cateter e fio guia até aorta abdominal. Depois, foi feita a passagem do microcatéter Rebar. Na artéria distal ao aneurisma, fez-se a liberação de micromolas Axium Frame de 10 x 40, 14 x 40 e 5 x 20 e a passagem do cateter balão Hyper Glide 5 x 20 para controle do fluxo sanguíneo com o agente embolizante Onyx™ 18 (3 mL, dois frascos). A angiografia de controle mostrou boa vedação na artéria distal. A seguir, foi passado um balão de 12 x 60 para oclusão temporária da artéria esplênica proximal ao aneurisma para facilitar a liberação das molas e do material embolizante e evitar a migração para dentro do saco aneurismático. Na artéria proximal ao aneurisma, por fim, fez-se a liberação de molas Axium Frame de 12 x 50, 6 x 20 e 5 x 20, micromolas Ruby Coil de 16 x 60, 8 x 60 e 4 x 35 e embolização com o agente Onyx (Figura 4).

**Figura 4:** Embolização do aneurisma maior (5,1 x 4,7 cm) pela técnica de *trapping*.

A angiografia de controle mostrou fluxo sanguíneo ausente para os aneurismas 1 e 2 e fluxo sanguíneo muito discreto passando pela artéria aferente ao aneurisma 3. Mesmo após várias tentativas com molas e material embolizante, optou-se por aguardar cessar o efeito da heparina e da possível trombose. A perfusão do pâncreas se manteve preservada, visto que havia perviedade das artérias pancreáticas dorsal e magna. Não houve infarto esplênico.

A paciente foi mantida na unidade de terapia intensiva para manejo clínico. Apresentou um hematoma em acesso braquial corrigido com arteriorrafia simples e não apresentou intercorrências no acesso femoral. Ela apresentou boa evolução no pós-operatório, mantendo sempre a necessidade de ajuste dos fatores de coagulação e plaquetas. Estava sem queixas de dor abdominal, recebendo alta no sexto dia de pós-operatório. No acompanhamento pós-operatório, a paciente permaneceu estável clinicamente, com melhora da função hepática, número de plaquetas e diminuição do baço. Fez acompanhamento ambulatorial para avaliação clínica e o ecodoppler de controle mostrou exclusão dos aneurismas 1 e 2 e trombose de 90% do aneurisma 3, com pequena zona de perfusão no colo próximo à artéria esplênica. Não houve crescimento desde o procedimento. Último exame realizado com quase 2 anos, manteve-se inalterado.

## DISCUSSÃO

A literatura mostra que os fatores de risco para o desenvolvimento do aneurisma de artéria esplênica incluem hipertensão arterial, doença aterosclerótica, cirrose, hipertensão portal, transplante de fígado, sexo feminino, gravidez e multiparidade^8^ . Entre esses fatores, a hipertensão portal é um fator de risco significativo para o desenvolvimento de aneurisma de artéria esplênica^9^ , sendo o risco de ruptura do aneurisma em pacientes com história de hipertensão portal maior que em pacientes sem essa história clínica (56% x 17%, respectivamente)^3^ . Em nosso caso, a paciente apresentava quase todos os fatores de risco citados acima. Por esse motivo, havia grande risco de ruptura do aneurisma e de infarto esplênico.

Embora se desconheça exatamente o mecanismo de dilatação aneurismática, alguns autores identificaram fatores contribuintes, como o enfraquecimento preexistente da parede arterial, a ruptura da lâmina elástica interna, a diminuição das fibras elásticas em multíparas e as alterações hormonais durante a gravidez. Essas condições podem estar presentes em pacientes com doença hepática e hipertensão portal com esplenomegalia, nos quais a incidência dos aneurismas de artéria esplênica é próxima de 20%^10^ .

É de grande importância ressaltar a possibilidade de ocorrerem complicações ao longo do tempo. Entre elas, a curto prazo, poderia ocorrer infarto esplênico, mas isso não ocorreu, pois a paciente apresentava boa circulação colateral pelas artérias gástricas curtas. Segundo a literatura, o infarto esplênico e a reperfusão do aneurisma ocorrem em 5 a 20% dos pacientes^11,12^ . A médio prazo, poderia ocorrer abscesso esplênico, e outra complicação possível seria a necrose pancreática, que, em nosso caso, a arteriografia de controle ao final do procedimento mostrou perviedade das artérias pancreática dorsal e magna, demonstrando boa perfusão do pâncreas. Para avaliar a reperfusão do aneurisma, é necessário acompanhamento anual por meio de tomografia computadorizada ou ressonância magnética de abdome^11^ .

As intervenções incluem cirurgia aberta tradicional, cirurgia laparoscópica e técnica endovascular. São recomendadas para pacientes com aneurismas sintomáticos, mulheres em idade fértil com aneurismas e aneurismas assintomáticos com 3 cm ou mais^7^ . Os procedimentos cirúrgicos incluem a ligadura ou ressecção das lesões proximais ou na porção média da artéria esplênica. A cirurgia de *bypass* não é necessária, uma vez que as artérias gástricas curtas fornecem um fluxo sanguíneo colateral para perfusão distal do baço. Por outro lado, a técnica de *trapping* endovascular, fornece uma alternativa minimamente invasiva em relação à ligadura cirúrgica. Nesse procedimento, o aneurisma é aprisionado e excluído da circulação ao serem colocadas molas na artéria esplênica distal e proximal ao aneurisma, certificando-se de que não permaneceu fluxo sanguíneo colateral para o aneurisma^6^ .

Os aneurismas de artéria esplênica costumam ser ideais para embolização devido ao suprimento colateral das artérias gástricas curtas. As taxas de sucesso relatadas variam de 90 a 100%^12-14^ . Para o aneurisma de artéria esplênica proximal e de vasos médios, podem ser utilizadas endopróteses para manter a perfusão na artéria principal; no entanto, a tortuosidade da artéria esplênica pode complicar a colocação e a implantação da endoprótese^12,15^ .

## CONCLUSÃO

Relatamos o caso de uma paciente de 52 anos, com cirrose, hipertensão portal, hiperesplenismo e apresentando múltiplos aneurismas de artéria esplênica. Portanto, a técnica endovascular foi uma boa opção devido às comorbidades e às discrasias sanguíneas apresentadas. Neste caso, o procedimento foi bem-sucedido. Apesar do sangramento no braço, a paciente teve boa recuperação e se manteve estável nos dois anos de acompanhamento. A embolização endovascular foi uma boa opção para os aneurismas 1 e 2 e a técnica do “trapping” endovascular teve resultado satisfatório no aneurisma número 3.
